# Stereo-electro-encephalography (SEEG) methodology: technical nuances and insights into image-guided robot-assisted electrode implantation

**DOI:** 10.3171/2024.4.FOCVID2427

**Published:** 2024-07-01

**Authors:** Piergiorgio d’Orio, Pedro Monteiro, José I. Otayza, Alessandra Rocca, Martina Revay, Laura Castana, Francesco Cardinale

**Affiliations:** 1 “Claudio Munari” Epilepsy Surgery Centre, Azienda Socio Sanitaria Territoriale Grande Ospedale Metropolitano Niguarda, Milan, Italy;; 2 Department of Medicine and Surgery, Unit of Neuroscience, University of Parma, Parma, Italy;; 3 Neurosurgery Department, University Hospital Center, Coimbra, Portugal;; 4 Hospital Barros Luco, Santiago, Chile; and; 5 Department of Medicine and Surgery, University of Milan Bicocca, Milan, Italy

**Keywords:** stereoelectroencephalography, drug-resistant epilepsy, robot-assisted surgery, image-guided neurosurgery, technical nuances

## Abstract

An accurate definition of the epileptogenic zone is critical to the success of epilepsy surgery. When noninvasive presurgical studies are insufficient, stereoelectroencephalography (SEEG) becomes indispensable. This study illustrates a systematic approach using an illustrative case of centroparietal epilepsy, detailing the stepwise workup, planning, and image-guided robot-assisted frameless stereotactic implantation of intracerebral electrodes. The video provides insights into technical aspects and a single-center experience. Demonstrating efficacy, safety, and feasibility, SEEG emerges as a valuable procedure for studying drug-resistant focal epilepsy.

The video can be found here: https://stream.cadmore.media/r10.3171/2024.4.FOCVID2427

## Transcript

SEEG was introduced in the 1950s at Sainte-Anne Hospital in Paris by Jean Talairach and Jean Bancaud.^1^ It is a methodology that allows 3D exploration of brain regions capable of generating seizures, thus better understanding how seizures begin and spread. This provides a better comprehension of the patient’s epileptogenic zone (EZ), defined as the site of the beginning of the epileptic seizures and of their primary organization.

### 0:51 Indications.

SEEG is indicated when noninvasive presurgical study fails to define patient’s EZ because anatomo-electro-clinical correlations appear discordant. It is very useful to study the relationships that the EZ may have with the eloquent areas. It can also provide information about the patient’s postsurgical prognosis and perform a proper risk-benefit assessment.^2,3^

### 1:15 Clinical Case.

In this video, we present our surgical workup through the presentation of an illustrative clinical case. This is a case of a 50-year-old male patient whose noninvasive assessment allowed us to presume a cryptogenic right parietal lobe epilepsy.

### 1:31 Image Acquisition.

All patients are imaged by a 3D MRI and 3D cone-beam CT digital subtraction angiography (3D CBCT DSA) in frameless and marker-less conditions, days or weeks before implantation. In our center, traditional Talairach technique has been progressively updated. Since 2009, we adopted an image-guided surgery workflow fully based on 3D imaging and robotic assistance (3D IRA).^4,5^

### 1:57 Planning.

Stereotactic trajectories are planned with Voxim (Renishaw Mayfield SA). Entry points and target points were defined for each trajectory by inspecting multiplanar reconstruction, brain, and vasculature surface rendering.

In our experience, 3D angiography is used as the stereotactic space where coordinates of entry and target points are stored. Because of its reliability, it is possible to check also for the smallest vessels, thus increasing safety.

Planned trajectories could be reformatted according to the planned vector. Because most hemorrhagic events originate mainly at the entry point, we usually set a radius of safe entry region of 2 mm, at least along the first third of the trajectory.^4^ Furthermore, it is useful to avoid trajectories too tangent to the skull surface. The quality and thickness of the bone itself have to be considered. In this case, a right centroparietal exploration was planned with the aim of also checking for possible seizure involvement of the sensory-motor cortex.

### 2:54 Surgery.

Surgery consists in an image-guided and robot-assisted electrode implantation.

### 2:58 Registration.

The procedure is performed with the patient under general anesthesia on supine position, being the head in neutral position. The disinfection of the entire scalp is performed before the head fixation.

The robotic system is then advanced toward the patient’s head with a sterile Leksell frame attached to it. The frame is used just to support the head, with no stereotactic purposes, as shown later in the video. The frame is fixed to the patient head, and the frameless and touchless patient registration procedure is started. Two skin fiducials were also placed, one on each side of the head. The neurolocate system (Renishaw Mayfield SA) is placed on the robotic arm and manually moved closer to the patient’s head. A cone-beam CT 3D scan with O-arm system is then acquired.^6^

A specific software module is used to complete the registration, selecting the center of the neurolocate’s fiducials in the multiplanar reconstructions provided by the Voxim stereotactic planning software. True trajectories pointing the skin markers are then planned in order to verify the registration. Finally, the planning software can compute the transformation matrix from the planning space to the robot space. Once the registration has been completed, the robotic arm can align the tool holder with the vector of the planned trajectories.

### 4:20 Screw Implantation.

The implantation starts by piercing the skin and subcutaneous tissue, with a very thin drill of 1 mm of diameter, which also performs a little indentation in the external cortical bone, as an "invitation" for the proper bone drill of 2.1 mm of diameter.

The bone is then drilled through its entire thickness without perforating the dura mater, this because the length of the drill was previously obtained subtracting the distance between robot tool holder and trajectory target point, minus the distance between the inner cortical layer of the bone and the target point itself.

The distance from the tool holder to the skin is measured, which leads to the choice of screw length subtracting this last measure to the drill length. The dura is coagulated and perforated with a monopolar coagulator. The appropriate screw is placed and the distance from the tool holder to the screw is measured.

Previously recorded distance from the tool holder to the target point, minus the distance from the tool holder to the screw will determine the length of the electrode. The same process is repeated for each screw.

Profuse washing of the hole with saline helps to remove any small bone fragments remaining. In a few cases, a cap can be placed to prevent excessive CSF leakage. Once all the guiding screws are fixed, the skin is disinfected and cleaned, then the frame is removed.

### 6:15 Electrode Implantation.

The relative position of each screw is recognized, and the electrodes can be inserted under radioscopic control.

First, a previously measured rigid stylet is placed and an x-ray image is obtained. Later, the electrode is introduced and another x-ray image is obtained. This technique is of utmost importance, as it not only creates the path for the electrode, which is semirigid, but also provides a position and depth reference to be compared in the radiographic image, obtained after the electrode is introduced. Electrode securing is achieved by tightening the cap to each screw.

Once all the electrodes are in place, a new intraoperative CT scan is acquired and coregistered to the preoperative planning in order to compare each electrode with its planned trajectory (yellow line).

### 7:10 Contact Verification.

Meanwhile, verification of proper electrode function is performed in the operating room by an epileptologist experienced in SEEG signal interpretation.

### 7:18 Surgical Dressing.

At the end, sterile draping is performed, surrounding all the screws with gauze, for isolation and comfort purposes, followed by bond dressing the head.

The patient is then awakened and transferred to the inpatient ward. The day after implantation, the patient is accepted to the recording laboratory for video-SEEG monitoring.

### 7:40 3D Reconstruction.

A multimodal scene is produced so that the team can recognize the electrodes and the location of each lead in the different brain structures explored.^7,8^

### 7:50 Video Monitoring Result.

Regarding this illustrative case, after 8 days of SEEG monitoring in both wakefulness and sleep, cortical stimulations, and neurophysiological assessments of sensory-motor areas, the conclusion was a complex nonlesional EZ involving the primary sensory cortex.

### 8:06 Electrode Removal.

After all useful data are obtained to define the epileptogenic zone, electrode removal is performed in the patient’s bedside (an exception is made for children, for whom electrode removal is done in the operating room under sedation).

After removal of the head dressing, the gauze is removed in a sterile manner, exposing the screws and electrode caps. The electrodes are detached from the screws and carefully removed, verifying their integrity, after which the screws are removed from the skull using available tools from the different electrode companies. After all electrodes and screws are removed, skin brushing is performed to occlude any communication with the intracranial space. If necessary, a simple skin seam with absorbable suture is applied. A new head dressing is placed, and if there are no complications, clinical or at the follow-up CT scan of the brain performed after this last procedure, the patient is usually discharged the next day.

### 8:57 Results.

We recently published our single-center experience. From 1996 to 2018, it has been performed 742 implantations, 43.5% of them with this 3D IRA workflow. Five hundred twenty-four patients underwent resective or disconnective surgery, and 53.2% of them are in Engel’s class I. Overall complication rate was 1.8% with better safety profile of 3D IRA workflow.^9^ Good procedural accuracy has been demonstrated in another recent paper of our group.^6^

### 9:29 Update.

To date, we have performed 970 implantations. According to the criteria of our last publication, there were 4 more complications, being 2 infectious, 1 TMJ dysfunction, and 1 electrode breaking, that needed subsequent surgery. No major complications were registered in the last 4 years.

### 9:46 Conclusions.

SEEG is a safe and effective procedure for the invasive assessment of the epileptogenic zone. The electrodes’ trajectories are carefully planned individually for each patient in order to gather information from the regions of interest, through their sampling, and the possibility to perform cortical stimulations. Well-defined steps, neuroimaging multimodal planning, and robot-assisted surgery increase the accuracy and safety of the procedure. Finally, postoperative 3D reconstruction of electrodes provides neurophysiologists a tool for better interpretation of the EZ’s spatiotemporal dynamic.

## Disclosures

The authors report no conflict of interest concerning the materials or methods used in this study or the findings specified in this publication.

## Author Contributions

Primary surgeon: Castana, Cardinale. Assistant surgeon: Monteiro, d’Orio, Revay. Editing and drafting the video and abstract: Monteiro, d’Orio, Otayza, Rocca. Critically revising the work: Monteiro, d’Orio, Rocca, Revay, Castana, Cardinale. Reviewed submitted version of the work: Monteiro, d’Orio, Castana. Approved the final version of the work on behalf of all authors: Monteiro. Supervision: d’Orio, Revay, Cardinale.

## Supplemental Information

### Patient Informed Consent

The necessary patient informed consent was obtained in this study.
